# A 1.8 mm-Diameter Chip-on-Tip Endoscope with Integrated µLED Illumination for Narrow-Cavity Imaging

**DOI:** 10.3390/s26144544

**Published:** 2026-07-17

**Authors:** Manuela P. Sá, Bernardo S. Dores, José A. Rodrigues, José H. Correia, Marino J. Maciel

**Affiliations:** 1 CMEMS-UMinho, University of Minho, 4800-058 Guimarães, Portugal; pg56141@alunos.uminho.pt (M.P.S.); bernardo.dores@cmems.uminho.pt (B.S.D.); jrodrigues@dei.uminho.pt (J.A.R.); mmaciel@dei.uminho.pt (M.J.M.); 2 LABBELS—Associate Laboratory, Braga/Guimarães, Portugal

**Keywords:** ultracompact endoscope, CMOS sensor, micro-LED, optical system, imaging performance, ex vivo validation

## Abstract

Advances in microelectronics have driven the miniaturization of endoscopic systems, leading to increasingly compact devices with enhanced optical and electronic performance. However, the current state-of-the-art commercial endoscopes—typically ranging from several millimeters to over 1 cm in diameter—still face significant limitations when accessing delicate anatomical structures such as the Fallopian tubes, urethra, and other narrow cavities. In this paper, we introduce an ultracompact endoscope based on the use of a CMOS image sensor from ams OSRAM (NanEyeM) and a single micro-LED (LTW-FC03DCD5). We achieved a total diameter of 1.8 mm, compatible with ultra-narrow cavities of the human body. Optically, a minimal correlated color temperature of 10,343 K and a maximal color rendering index of 73.6 were achieved. These values allow for the detection of structures of small dimensions by morphological and vascular contrast. The optical system demonstrated the ability to resolve up to 83 lp/mm, with a diagonal field-of-view of 115.0° ± 2.4°, measured at different working distances, suitable for working distances encountered in narrow anatomical cavities. This work represents a first prototype for direct visual imaging in human organs not previously accessible by endoscopy, assisting healthcare professionals in clinical assessment and supporting the diagnosis of diseases.

## 1. Introduction

Endoscopy has transformed diagnostic and therapeutic practices across multiple medical specialties, allowing for minimally invasive interventions that reduce trauma, recovery time, and postoperative complications [1,2,3,4]. These specialties include gastroenterology, urology, otorhinolaryngology, gynecology, and pulmonology [5,6]. Current flexible endoscopes typically exhibit outer diameters ranging from approx. 2.9 mm for transnasal models to 15 mm for gastrointestinal endoscopes. By contrast, rigid endoscopes generally have outer diameters between 5 and 12 mm for laparoscopic applications and between 2 and 4 mm for neurosurgical or nasal endoscopy [5,7,8]. These dimensions severely limit access to narrow and delicate anatomical structures, such as the Fallopian tubes, fimbria-ovary junction, urethra, and other luminal organs with characteristic diameters of approx. 1 mm. Consequently, these regions remain inaccessible to direct endoscopic visualization [9].

The need to access ultranarrow cavities is particularly critical in gynecology, since the Fallopian tubes are recognized as a potential site of origin for ovarian cancer [10,11]. Given that this disease represents the 8th leading cause of cancer-related death among women and about 5% of female cancer deaths worldwide [12], the development of endoscopes capable of navigating openings smaller than 2 mm would represent a crucial advance for early diagnosis.

The evolution of endoscopic imaging has been strongly driven by advances in image sensor technology. The introduction of digital imaging with charge-coupled device (CCD) sensors in the mid-1980s represented a major breakthrough, enabling sensor miniaturization and allowing the entire imaging process to take place at the distal tip of the endoscope [13]. With the transition to the 21st century, developments in complementary metal–oxide–semiconductor (CMOS) image sensors drove a decisive transformation in endoscopic engineering. CMOS sensors offer higher levels of electronic integration, lower power consumption, on-chip signal processing and reduced thermal noise, while enabling further miniaturization without compromising optical sensitivity [14,15,16]. The technological transition from CCD to CMOS sensors was the decisive driver for extreme miniaturization.

Endoscopic illumination systems have progressed in parallel, leading to the establishment of two principal approaches currently used: external light sources coupled with fiber-optic transmission and direct LED-on-tip illumination [17]. One of the biggest obstacles to reducing the overall diameter of the endoscope is the lighting system [18]. External light sources coupled with fiber optic bundles are bulky and expensive [17,18]. Integrating LED lighting at the distal end (tip) of the device not only reduces the final diameter to levels below 2 mm but also ensures the economic viability of single-use devices, eliminating the need for labor-intensive reprocessing and complex optical components [19].

Despite substantial miniaturization efforts and enhanced functional capabilities, commercial endoscopes remain relatively large for accessing narrow anatomical structures. Ongoing work still confronts several core limitations: achieving uniform illumination at extremely small diameters, preserving high spatial resolution, and enabling high-speed, real-time video imaging within ultra-miniature cavities. In this context, we introduce a compact endoscope with a 1.8 mm diameter, designed with the ultimate goal of visualizing narrow cavities that have never previously been accessible by imaging. This endoscope integrates a CMOS image sensor (NanEyeM, ams-OSRAM, Premstaetten, Austria) [20] and a single micro-LED (µLED) (LTW-FC03DCD5, LITEON, Taipei, Taiwan, China) [21], both mounted at the endoscope tip. The prototype incorporates single-axis angular oscillation, enabling real-time internal access over an extended field of view within hollow, narrow organs such as the Fallopian tube. This work represents one of the first steps toward endoscope miniaturization for gynecological applications.

## 2. Materials and Methods

### 2.1. Prototype Design

The proposed device is a proof-of-concept miniaturized endoscopic prototype that aims to provide access to and visualization of anatomical structures that remain inaccessible with conventional endoscopic instruments. The system was developed to investigate the feasibility of sub-2 mm diameter endoscopes for applications requiring direct access to and detailed observation of the Fallopian tubes, urethra, and smaller-caliber vasculature, where stringent dimensional constraints and enhanced navigational capability are essential.

The overall system architecture is based on the integration of four primary functional modules: a distal imaging unit incorporating a tip-mounted CMOS camera; an illumination module consisting of a µLED positioned at the distal end; a steering mechanism enabling lateral motion of the endoscopic tip; and a flexible body that supports insertion and navigation through confined anatomical pathways. This distal integration strategy enables in situ image acquisition at the point of interest while minimizing the optical losses typically associated with proximal imaging configurations.

The prototype was designed to achieve an overall outer diameter of less than 2 mm at the distal end, encompassing all integrated components. This dimensional constraint dictated the spatial arrangement and packaging strategy of the imaging, illumination, and actuation elements. A compact commercial CMOS camera with lateral dimensions of approximately 1.0 × 1.0 mm^2^ was selected as the imaging core (NanEyeM, ams-OSRAM, Premstaetten, Austria) [20], enabling direct tip-mounted acquisition while preserving a minimal footprint. The µLED (LTW-FC03DCD5, LITEON, Taipei, Taiwan, China) [21] was positioned in proximity to the camera at the distal end to provide localized illumination of the field of view, ensuring adequate scene brightness within the confined anatomical environment. The distal assembly was designed with a modular approach, in which a rigid housing accurately positioned and secured both the CMOS sensor and the illumination source. This housing was designed to maintain fixed relative alignment between components, which is critical for consistent illumination and image quality. Design priority was placed on minimizing obstruction of the camera’s field of view and maximizing illumination uniformity, resulting in a lateral offset configuration between the µLED and the imaging axis.

The steering mechanism employs a fine stainless-steel wire (50 µm diameter) routed through a support feature, facilitating mechanical actuation from the proximal to the distal end. Tension on the wire induces lateral deflection of the distal segment, allowing precise, 1 axis lateral orientation of the viewing angle. This method was chosen for its simplicity and compatibility with dimensional constraints. A flexible encapsulation couples the distal imaging and lighting module with the steering module, using a flexible material to maintain compliance and structural integrity.

Overall, the design prioritizes compact integration, mechanical robustness, and functional modularity, establishing a platform suitable for evaluating the feasibility of ultra-miniaturized endoscopic systems.

### 2.2. Fabrication of the Imaging and Illumination Module

**Figure 1:**
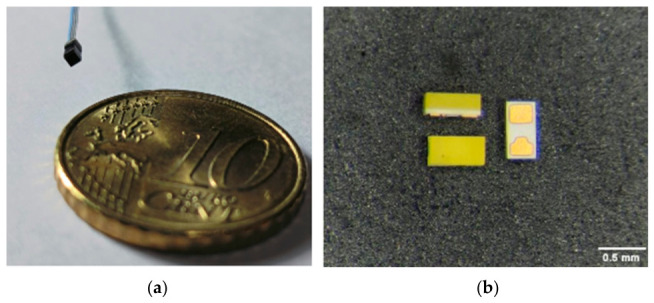
(**a**) NanEyeM camera module next to a 10-cent coin for scaling; (**b**) front, back, and side views of the µLED.

The choice of image sensor is critical, as it directly impacts the device’s final diameter, imaging capabilities, and the viability of integrating it into a chip-on-tip architecture. For these reasons, the NanEyeM micro-CMOS camera (NanEye series, ams OSRAM) [20] was selected as the prototype’s image core, due to its small footprint and suitability for minimally invasive endoscopic applications, as observed in Figure 1a. The sensor was designed primarily for systems where dimensional constraints are critical, while still retaining the capability to transfer image data across long connector lengths, making it ideal for small and slender endoscopic modules.

The camera module has lateral dimensions of 1.0 × 1.0 mm^2^ and a total length of 2.77 mm, allowing integration with lighting components within a distal diameter of approximately 2 mm. The sensor’s image matrix has a native resolution of 320 × 320 pixels and a pixel size of 2.4 µm × 2.4 µm. In addition to its compact form factor, the sensor integrates a 10-bit ADC and a serial LVDS output interface, enabling direct digital picture transfer while reducing the number of required interconnections. The sensor also supports programmable exposure duration, analog gain, dark-level adjustment, and frame-rate control, enabling adaptation to varying lighting conditions during experimental assessment. Another important consideration in camera selection is the optimization for medical endoscopic applications, namely signal-to-noise ratio performance and compatibility with small imaging assemblies. The capacity to transmit signals over cables up to several meters (2.5 m) facilitates future incorporation into extended flexible endoscopic systems.

For distal illumination, the Lite-On LTW-FC03DCD5 [21] white µLED, Figure 1b, was selected for its compatibility with the dimensional, optical, and electrical requirements of the proposed miniature endoscopic prototype. It has an ultra-compact footprint of 0.6 × 0.3 × 0.2 mm, enabling its integration next to the camera module, while keeping the entire distal section diameter below 2 mm. This compact footprint was a key consideration in the selection of this µLED as the illumination component of the prototype.

From an electrical and thermal perspective, the µLED runs at low, forward currents, typically about 5 mA, and forward voltages ranging from 2.7 to 3.1 V, leading to low power consumption and less heat production, important characteristics for endoscopic systems that are designed for use near biological tissue where excessive heat dissipation must be avoided. As for the LED’s optical emission properties, it has a broad viewing angle of around 120°, which coincides exactly with the field-of-view (FOV) of the NanEyeM camera and is extremely useful for short-range intraluminal imaging where the distance between the light source and the target region is small. The wide illumination angle promotes more uniform coverage of the camera’s FOV and reduces localized intensity gradients, thereby improving image visibility.

The structure was designed using Autodesk Fusion 360, v.2605.1.52, and fabricated using stereolithography-based resin 3D printing with an Anycubic Photon Mono 4K printer, v0.0.11 (Shenzhen Anycubic Technology Co., Ltd., Shenzhen, China). White photopolymer resin was used over clear resin to prevent internal light dispersion and optical interference between the lighting system and the CMOS sensor. Following manufacturing, the printed components were washed using isopropyl alcohol (IPA) to remove any remaining non-polymerized resin, and then post-cured under UV light for 5 min to complete polymerization and increase mechanical stability.

The early iterations of the distal support featured a modular two-part design, with the top part securing the µLED and the lower part housing the CMOS camera module. This design enabled the optimization of the alignment between the illumination and imaging systems. Modifications explored included variations in CMOS camera and µLED housing tolerances, the number of integrated µLEDs, and the thickness of a resin layer between the µLED housing and the emission surface. The addition of 100 µm resin layers aimed to enhance light diffusion, improving uniformity and reducing harsh lighting effects. Initial assembly relied on inserting the camera body into the support, but issues with mechanical stability led to the incorporation of locking elements for improved robustness during handling.

**Figure 2:**
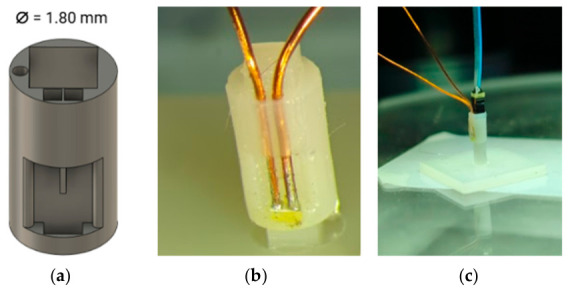
Assembly of the imaging and illumination module: (**a**) 3D model; (**b**) soldering of the 200 µm copper wire to the µLED pads; and (**c**) insertion of the camera into the module.

The early prototypes had an exterior diameter of around 2.0 mm. Following assembly tests, an improved, one-part-only version with a diameter of 1.8 mm was created, in line with the miniaturization objectives. Apart from being a one-part piece, this last prototype had other improvements, such as a side opening to support the insertion of the µLED and individual wire channels to guide and align the electrical wires precisely with the µLED electrical pads Figure 2a. The µLED, CMOS camera, and electrical linkage connections could now be integrated into a single mechanical structure, improving assembly longevity and structural integrity. A significant challenge in prototype development was integrating the electrical connections at the distal end of the endoscope, due to the compactness of the components and the severe spatial constraints imposed by the miniature form factor. Initial approaches using wire bonding for the µLED connections proved inadequate and mechanically frail. This was addressed through manual soldering with 200 µm copper wire connections, as observed in Figure 2b, enhancing both electrical reliability and mechanical strength. A mounting jig was also fabricated to assist with the assembly.

Once fabricated, the single part was positioned in the mounting jig with the distal plane facing downwards. The µLED was then inserted through the side opening and pressed into its housing with the soldering pads facing upwards; the copper wires were each inserted through their individual channel and subsequently soldered to the µLED pads. These features—secure prototype fixation within the jig, individually routed wire channels, and a delicate side opening—collectively contributed to a rapid and repeatable assembly process. The printed single part was slightly raised to guide the camera module into position, after which it was removed from the jig and the camera fully inserted, see Figure 2c. It is important to mention that after the wires were soldered, a small drop of resin was placed in the side opening to fill it and secure the wires in place.

### 2.3. Fabrication of the Steering and Flexible Module

**Figure 3:**
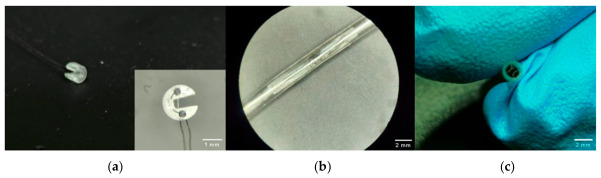
(**a**) Image of the steering module with the stainless-steel pull wires inserted; (**b**) image of the flexible encapsulation tube; (**c**) image of the pull wire’s dedicated channels to prevent twisting.

For the steering module, an additional structure was developed to interface with the distal imaging and illumination module of the endoscopic device, enabling controlled lateral deflection of the endoscopic tip. The structure, which is positioned right behind the imaging and illumination assembly, has the same outer diameter of 1.8 mm and includes: a central longitudinal groove that guides electrical connections from the CMOS camera and µLED; two identical 400 µm channels in opposite side for the 50 µm stainless steel wires; and a peripheral groove connecting both channels on the front of the structure to facilitate assembly, as presented in Figure 3a.

The assembly procedure is as follows: the stainless-steel wire is inserted at the back end of the module through one of the channels and guided towards the front end, where it exits the structure; it is then routed through the second channel from the front to the back and tensioned until slack at the front end is seated in the peripheral groove.

The steering structure was designed and fabricated using the same process as the imaging and lighting module.

After the steering structure fabrication, a flexible encapsulation tube was developed to mechanically couple the steering module to the distal imaging and lighting module, as seen in Figure 3b. This component was fabricated using flexible photopolymer resin, specifically the Inslogic Flexible 70A Resin (Inslogic, Hong-Kong, China), allowing localized bending of the distal segment under wire actuation while maintaining structural continuity between the rigid sections of the prototype. The flexible tube includes dedicated 650 µm channels for the pull wires to go through, preventing them from twisting inside (Figure 3c), and was designed with a nominal outer diameter of 2.05 mm, oversized relative to the target dimensions to account for shrinkage during the printing and post-curing process. The use of a compliant encapsulation material further contributed to reducing stress concentration during bending and enhancing the mechanical robustness of the assembled structure during manipulation and testing.

With both modules already installed inside the encapsulation tube, a standard 2 mm transparent heat-shrink tube was applied over the remaining length of the endoscope, from the proximal end of the encapsulation tube to the handle entrance, and retracted with a heat gun under slight longitudinal tension, achieving a controlled maximum outer diameter of 1.75 mm.

### 2.4. Prototype Assembly

With all the materials and modules designed and fabricated, the final step was to assemble all components.

Firstly, both pull wires of the steering mechanism were inserted through their dedicated channels in the flexible encapsulation tube, allowing them to be pulled from the proximal end to insert the 3D-printed part into the tube. In the second step, the camera and µLED wiring from the imaging and illumination module were pulled through the existing groove on the steering mechanism component, already seated within the flexible tube, and carefully tensioned to position the distal module directly above the steering module. The third and last step consisted of introducing the shrinking tube over the flexible one, sliding it toward the proximal end of the tube, and shrinking it with a heat gun to secure both tubes together and achieve the desired outer diameter.

The fully assembled prototype has a measured maximum outer diameter of 1.93 mm at the distal rigid tip over a length of 8 mm, tapering to 1.75 mm along the proximal shaft. The total deflectable distal section has a length of 16.3 mm, and the total inserted length is 40 cm. Table 1 summarizes the time required to fabricate and assemble the different components and modules. In total, the time to assemble the prototype from start to finish is roughly 5 h and 50 min.

**Figure 4:**
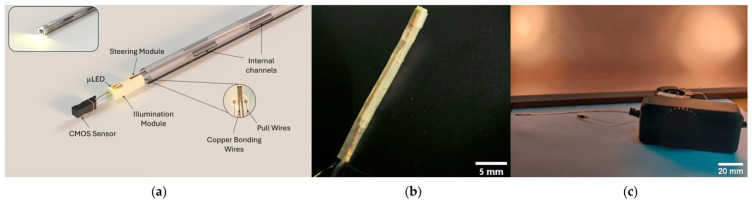
(**a**) Exploded 3D rendering of the prototype components prior to assembly; (**b**) distal part of the assembled prototype, with the image and illumination module, steering module and encapsulation tube together; (**c**) prototype attached to the 3D printed handle with the shrinking tube.

An exploded 3D rendering of the prototype components is presented in Figure 4a. With the prototype assembled, as shown in Figure 4b, it is finally attached to a 3D-printed handle, with the pull wires fixed to a wheel to control the endoscope’s distal end motion. The final mounting is shown in Figure 4c.

### 2.5. Illumination System Characterization

The prototype was experimentally characterized through a series of optical, electrical, and mechanical measurements aimed at evaluating the performance of its three main subsystems: the illumination module, the imaging system, and the distal tip actuation mechanism.

All measurements were performed at room temperature, and during optical characterization, the µLED was driven using a calibrated current source (Yokogawa 765, Yokogawa Manufacturing Corporation, Yamanashi, Japan) to ensure stable and repeatable operating conditions.

#### 2.5.1. Spectral Properties: Spectral Power Distribution (SPD), Correlated Color Temperature (CCT), and Color Rendering Index (CRI)

**Figure 5:**
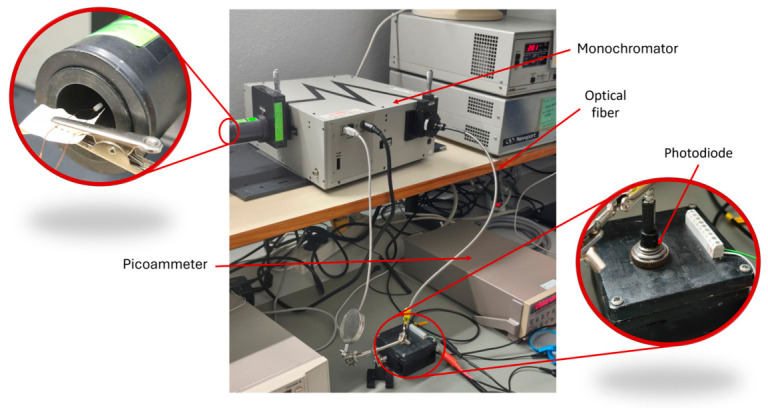
SPD acquisition setup, consisting of a Newport 77524 optical fiber, a Newport 74125 monochromator, the Hamamatsu S1336-5BQ photodiode, and a Keithley 487 picoammeter.

The SPD of the µLED was acquired by coupling the light emitted at the distal tip of the prototype into an optical fiber (Newport 77524, Newport Corporation, Irvine, CA, USA) connected to a monochromator (Newport 74125), which scanned the visible range from 380 to 780 nm in 1 nm steps. The intensity at each wavelength was detected using a silicon photodiode (Hamamatsu S1336-5BQ, Hamamatsu Corporation, Hamamatsu city, Japan), and the resulting photocurrent was measured using a picoammeter (Keithley 487, Keithley Instruments, Inc., Solon, OH, USA). Data acquisition was performed in a dark room to prevent contamination of the measured signal from the ambient light. Measurements were conducted at five drive-current levels (2.0, 5.0, 10.0, 15.0, and 20.0 mA), covering the full operating range of the µLED. The acquisition setup can be seen in Figure 5.

A baseline correction was applied during post-processing by subtracting the mean signal of the first 10 wavelength samples (380–389 nm), which lie outside the µLED emission band and therefore represent the background level, from each acquisition.

For each baseline-corrected SPD, the CCT and the general CRI (CRI Ra) were computed in Python, v.3.10.20, using the LuxPy toolbox [22], which implements the procedure standardized in CIE 13.3-1995, as described in detail in [23]. The CCT was obtained by converting the SPD into CIE 1931 (x, y) chromaticity coordinates and then into the CIE 1960 UCS (u, v) space, where the closest point on the Planckian locus is identified using the parabolic interpolation method proposed by Ohno [24]. The CRI Ra was determined by simulating the appearance of eight Munsell test color samples (TCS1 to TCS8), whose spectral reflectance is listed in CIE 13.3-1995, under illumination by both the µLED and a reference light source of matched CCT. For each sample, the color difference between the two illumination conditions defines an individual rendering index Ri, and Ra is computed as the average of these eight values.

The chromaticity coordinates (x, y) and the distance to the Planckian locus (Δuv) were also extracted to track the chromatic stability of the illumination across the operating range. In endoscopic applications, this stability is relevant because shifts in color appearance can compromise image consistency and reduce the perceived contrast of the tissue.

#### 2.5.2. Light-Current-Voltage (LIV) Behavior

**Figure 6:**
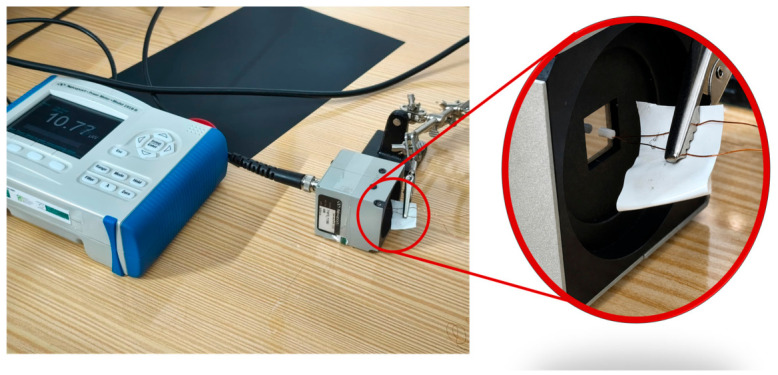
LIV acquisition setup, consisting of a Newport 1918-R power meter, a Newport 71580 optical detector, and the µLED.

The optical power and forward voltage of the µLED were characterized as a function of the drive current. The optical power emitted at the distal tip was collected by a calibrated optical detector (Newport 71580) connected to a power meter (Newport 1918-R), which provided the optical power readings. The acquisition setup can be seen in Figure 6. Simultaneously, the forward voltage across the µLED terminals was recorded using a digital multimeter (LG DM-3111, LG Precision Co. Ltd., Seoul, South Korea), under dark-room conditions. The drive current was swept from 0.5 to 20.0 mA in increments of 0.5 mA, remaining within the maximum forward current specified in the manufacturer’s datasheet (LTW-FC03DCD5) [21].

From the LIV measurement, the electrical input power (*P*, in Watts) was calculated as:

(1) $$P=V_{F}\cdot I_{F},$$

where $V_{F}$ is the forward voltage (V) and $I_{F}$ is the forward current (A). The wall-plug efficiency (*WPE*), defined as the fraction of electrical power, $P_{el}\;$, converted into optical power, $P_{opt}$, was obtained as the ratio of optical power to electrical input power:

(2) $$WPE=\frac{P_{opt}}{P_{el}},$$

The following slope efficiency:

(3) $$\eta_{s}=\frac{dP_{o_{P}t}}{dI},$$

was extracted by applying a linear fit to the linear portion of the L-I curve. The upper limit of this interval was chosen below the onset of efficiency droop, a well-known reduction in efficiency observed in nitride-based LEDs at high drive currents [25], where the optical output starts to increase sub-linearly with current. The lower limit was defined above the low-current region, where the L-I response is not yet linear.

#### 2.5.3. Irradiance Characterization

**Figure 7:**
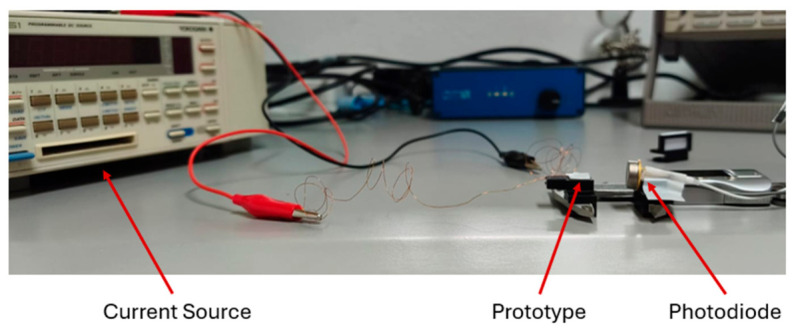
Irradiance acquisition setup, consisting of the Yokogawa 765 current source, the prototype illumination module, and the Hamamatsu S1336-8BK photodiode, aligned on a digital caliper for precise and repeatable measurements.

The irradiance produced by the prototype was characterized as a function of both the µLED drive current and the working distance expected during navigation in narrow anatomical cavities. For this purpose, the prototype distal tip was aligned directly with a photodiode (Hamamatsu S1336-8BK, Hamamatsu Corporation, Hamamatsu city, Japan), and the generated photocurrent was recorded. To ensure controlled and repeatable adjustment of the working distance, a custom 3D-printed holder was developed to mount both the prototype and the photodiode on a digital caliper. As can be seen in Figure 7, each component was fixed to one of the jaws of the caliper, enabling direct assessment of the separation between the µLED and the detector’s active area.

Measurements were acquired at working distances of 5.0, 10.0, 20.0, 30.0, and 40.0 mm, while the µLED drive current was swept in 1.0 to 20.0 mA increments. All readings were performed under dark conditions, and for each acquisition, a corresponding dark-current value was obtained with the µLED switched off, while maintaining the rest of the setup unchanged. This background signal was subtracted from the measured photocurrent to minimize the contribution of residual ambient light and detector dark noise.

To obtain the optical power reaching the photodiode from the corrected photocurrents, an effective responsivity, Reff, was used as a calibration factor specific to this µLED. Since the spectral responsivity of the photodiode, *R*(*λ*), varies with wavelength, and the µLED does not emit uniformly across the visible range, *R*(*λ*) was averaged using the normalized emission spectrum of the LED *S*(*λ*) as a weighting function:

(4) $$R_{eff}=\frac{\int S(\lambda )R(\lambda )\;d\lambda }{\int S(\lambda )\;d\lambda }$$

Here, *S*(*λ*) is used only to describe the shape of the µLED emission, not the absolute intensity. *R*(*λ*) was obtained by digitizing the spectral responsivity curve provided by the manufacturer, with an estimated uncertainty of approximately 5%. *S*(*λ*) corresponds to the emission spectrum measured at 5.0 mA in Section 2.5.1, which is the typical drive current specified by the manufacturer. The resulting value, $R_{eff}$ = 0.320 A/W, was then used to recover the optical power reaching the photodiode from each photocurrent reading, following the standard formulation of broadband radiometric measurements with silicon photodiodes [26]. Finally, the optical power was divided by the active area of the photodiode to obtain the irradiance E (W/m^2^) at each combination of drive current and working distance.

**Figure 8:**
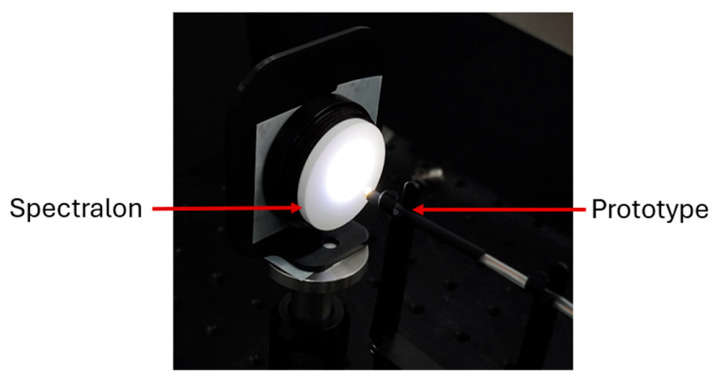
Illumination uniformity characterization setup, consisting of the prototype mounted on a Thorlabs optical table with alignment holders and directed at a 1-inch Spectralon diffuse reflectance standard.

The spatial uniformity of the illumination across the field of view (FOV) was assessed by placing a 1-inch Spectralon diffuse reflectance standard (Labsphere, Sutton, NH 03260, USA, reflectance ~99%) at the focal plane of the endoscope tip and imaging it directly using the prototype, following the approach described by Wang et al. [27]. The prototype and the Spectralon target were mounted on a Thorlabs optical table using dedicated alignment holders to ensure stable and repeatable positioning along the same optical axis, as shown in Figure 8.

Measurements were performed at working distances of 4 mm and 6 mm. The lower limit of 4 mm corresponds to the minimum working distance below which the imaging system cannot form a focused image. The upper limit of 6 mm was imposed by the diameter of the available Spectralon standard, beyond which part of the illuminated field falls outside the target surface.

For each working distance, 30 illuminated frames and 30 dark frames were acquired under two sensor operating conditions: the optimized fixed-gain configuration (5 mA, 120 µs, Gain = 1, Offset = 1) and automatic gain control. The acquired frames were averaged to reduce the contribution of shot and read noise. The averaged dark frame was subsequently subtracted from the averaged illuminated frame to compensate for dark current and fixed-pattern noise contributions, which are intrinsic properties of the sensor and independent of the illumination scene. The resulting corrected frames were then normalized to the maximum corrected intensity and to the diffuse reflectance factor of the Spectralon standard (ρ = 0.99), producing a relative irradiance map for each working distance and sensor configuration.

The illumination uniformity was quantified using the coefficient of variation [28,29], defined as:

$$CV=\frac{\sigma }{\mu }$$

where *σ* is the standard deviation, and *µ* is the mean of the relative irradiance values computed over the full 320 × 320-pixel array. A *CV* of zero corresponds to a perfectly uniform distribution, with higher values indicating greater spatial variation in the illumination intensity across the FOV.

### 2.6. Imaging System Characterization

#### 2.6.1. Field of View

The field of view (FOV) of the assembled imaging system was characterized under its normal operating conditions, using the integrated µLED at the distal tip to illuminate the scene. This approach allows the measured FOV to reflect the effective imaging region of the complete system, rather than the nominal camera FOV under ideal external illumination conditions.

**Figure 9:**
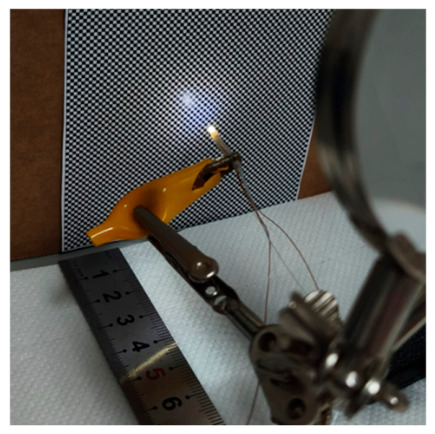
Experimental setup for field-of-view characterization of the endoscope prototype, using a planar checkerboard target (1 mm squares) at controlled working distances.

The imaging performance of the prototype was evaluated using a planar checkerboard target, following the method used by Fröch et al. [30], composed of 1 mm squares (80 × 80 mm). During the measurements, the target was positioned perpendicular to the optical axis, while the endoscope was mounted on a fixed support to ensure repeatable alignment and working distance, as can be seen in Figure 9. Images were acquired at controlled distances (6.0, 8.0, and 10.0 mm) using the NanEye Viewer, v6.15.3.7, with exposure time and analog gain adjusted manually and kept constant throughout the characterization.

The horizontal and vertical FOVs were determined from the number of complete squares visible in each direction of the image, by applying the trigonometric formulation reported by Wang et al. [31] for endoscope *FOV* characterization:

(5) $$FOV=2\cdot \arctan\left(\frac{N\cdot s}{2\cdot d}\right),$$

where *N* is the number of squares within the imaged area along the considered direction, *s* is the physical size of each square (1 mm), and *d* is the working distance between the endoscope tip and the pattern.

#### 2.6.2. Slanted-Edge Modulation Transfer Function (MTF)

For spatial resolution characterization, the MTF was measured at working distances of 2, 4, 6, 8 and 10 mm using the slanted-edge method following the ISO 12233 standard [32,33]. The imaging module was mounted on a three-axis micromanipulator to enable precise and repeatable control of the working distance. A razor blade was used as a knife-edge target, as it provides a straight, high-contrast edge. It was positioned at a small angle of approximately 12° relative to the sensor sampling grid to avoid aliasing artefacts, and placed in front of a 1 cm-thick acrylic diffuser plate uniformly illuminated from behind by a lamp, to provide diffuse backlighting, which is the recommended illumination geometry for slanted-edge measurements [27]. The MTF was extracted from the acquired images using MTF Mapper, v0.7.40, an open-source software that implements the slanted-edge pipeline: the edge spread function (ESF) was extracted from the knife-edge region, differentiated to obtain the line spread function (LSF), and subsequently Fourier-transformed to yield the MTF curve at each working distance. Measurements were performed under both fixed and automatic gain conditions to assess the influence of gain settings on the measured spatial frequency response.

The spatial resolution, expressed in lp/mm, was quantified from the MTF50 values obtained across all working distances, with the peak MTF50 reported as the best achievable resolving capability of the system.

### 2.7. Mechanical and Safety Performance

#### 2.7.1. Thermal Behavior in Tissue-Mimicking Phantom

The contact between the distal tip of the endoscope and biological tissue during navigation requires that any thermal effects of LED illumination on the surrounding tissue be quantified, as prolonged exposure to elevated temperatures can compromise tissue integrity. To evaluate this, the prototype’s temperature rise was measured in a phantom designed to simulate tissue under conditions like those anticipated during in vivo operation.

To reproduce conditions representative of soft tissue, a tissue-mimicking phantom was prepared using a 2.5% (*w*/*v*) agar gel in distilled water. Agar-based phantoms are widely used in thermal characterization studies because their behavior is dominated by water content, resulting in thermal properties comparable to those of soft biological tissues [34]. The agar powder was dissolved in water heated to approximately 97 °C under continuous stirring, after which the solution was transferred into a mold and left to solidify at room temperature.

**Figure 10:**
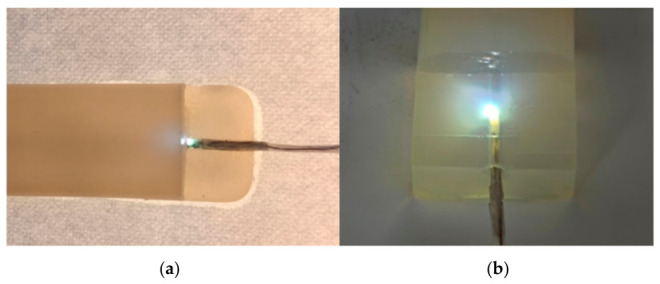
Thermal characterization using an agar-based tissue-mimicking phantom: (**a**) prototype placed on the phantom surface, (**b**) prototype inserted into a tubular phantom.

Two experimental configurations were tested to represent the most relevant interaction geometries: a surface configuration, Figure 10a, in which the distal tip of the prototype was placed in contact with the upper face of the phantom, and a confined configuration, Figure 10b, in which the endoscope was inserted into a thin lumen molded into the phantom, reproducing the geometry of a narrow anatomical cavity. For each configuration, the µLED was driven at 5.0 mA and 20.0 mA, the typical drive and maximum current specified by the manufacturer, respectively.

Temperature was monitored using an infrared thermal camera (Optris) directed at the phantom during prototype operation. Image acquisition was continuous throughout the test, allowing the full heating profile to be analyzed from the moment the µLED was switched on until a steady-state temperature was reached. The initial temperature of the agar was recorded at the start of each reading, and the temperature rise was calculated as the difference between the steady-state temperature and this initial value.

The measurements were performed at room temperature in a tissue-mimicking phantom. Although the thermal conductivity of the agar phantom is representative of highly hydrated soft tissues, physiological mechanisms such as blood perfusion were not replicated. The reported temperatures are an experimental estimate of the prototype thermal behavior, namely the temperature rise while using the µLED.

#### 2.7.2. Tip Contact Force and Wire Integrity

The mechanical behavior of the distal tip of the prototype was evaluated through three tests: the force exerted by the tip when actuated against a surface, the traction force required to deflect the prototype across its angular range, and the bending radius achieved at full deflection. These tests address both the device’s safety when in contact with soft tissue and its ability to navigate the curvature of the Fallopian tubes.

**Figure 11:**
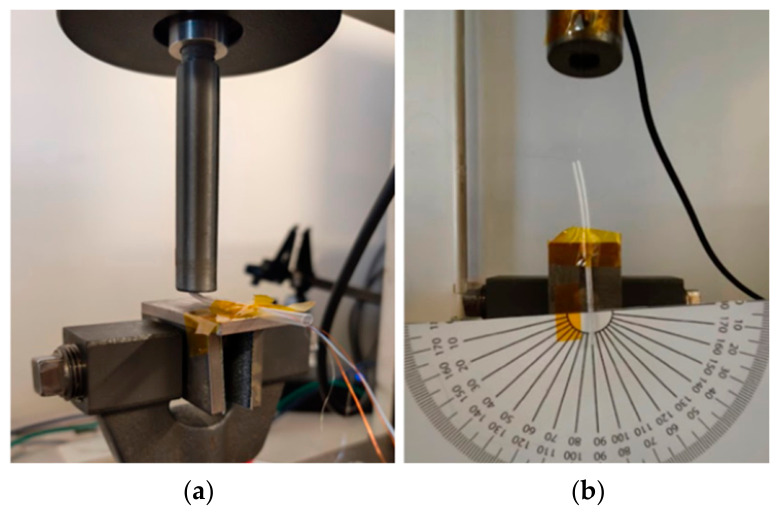
Mechanical tests performed on the endoscope prototype: (**a**) tip force measurement; (**b**) traction test.

The contact force generated by the distal tip during actuation was measured in a Shimadzu AG-IS dynamometer (Shimadzu Corporation, Kyoto, Japan) equipped with a 10 N load cell mounted above the prototype on a fixed support. The prototype was placed horizontally, as shown in Figure 11a, so that its distal tip rested against the base of the load cell without any preload, and tensioning the steering wire from the proximal end deflected the tip upward into the load cell.

The maximum force reached was taken from the acquired curve, and because the load cell blocks further deflection as soon as contact is established, this value represents the large force the tip can exert against a surface during actuation, rather than the force at a fixed deflection angle. Two prototype configurations were tested: a fully assembled device with the imaging and illumination module integrated and a prototype without the image and illumination system, allowing the influence of the distal module on force transmission to be assessed separately. The complete prototype was tested in two independent trials, while the bare prototype was tested once.

The traction force required to deflect the prototype was characterized in a separate setup, seen in Figure 11b, with the prototype held vertically and the proximal end of the wire clamped to a mechanism coupled to the same load cell. The wire was pulled upward at a controlled rate (1.0 mm/min) while the angular deflection of the tip was followed with an angle gauge placed under the prototype, allowing the traction force to be paired with the angle reached at each instant of the test. The measurement was carried out up to 60° of deflection, which corresponds to the steering angle reported by Cordova et al. [35] required to follow the curvature of the Fallopian tube.

The bending radius at full deflection was extracted from a lateral photograph of the prototype tractioned to the 60° position. Points along the centerline of the curved section were manually marked in ImageJ software, v1.54g. The image scale was set using the lateral dimension of the CMOS camera package as an in-image reference, converting the marked coordinates from pixels to millimeters. A circle was then fitted to those coordinates in Python by least squares, returning both the radius of the curvature and the root-mean-square error between the measured points and the fitted circle.

### 2.8. Ex Vivo Validation in Reproductive Tract Tissue

To check whether the system can capture the visual information needed for clinical use, the prototype was also tested in an ex vivo porcine reproductive tract, which offers anatomical structures comparable in scale and morphology to the human Fallopian tube and surrounding organs. The reproductive tract samples used were obtained from female pigs at a local slaughterhouse. After collection, the samples were separated and stored frozen until use and thawed overnight before imaging.

**Figure 12:**
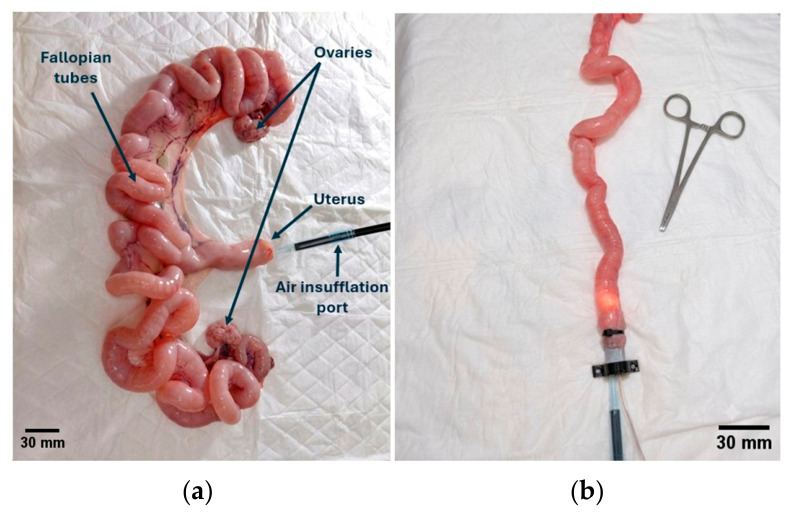
Setup for the ex vivo validation. (**a**) Porcine reproductive tract intact, where we can distinctly see the uterus, Fallopian tubes, and ovaries, inflated with compressed air; (**b**) Proximal portion of the Fallopian tubes, with a 3D printed support piece.

Two tissue configurations were used. In the first, shown in Figure 12a, the reproductive tract was kept intact, enabling navigation of the prototype through the uterine cavity and into the Fallopian tubes, in a configuration closer to the intended clinical scenario. In the second, Figure 12b, the Fallopian tubes and ovaries were dissected from the uterus and tested as isolated structures, allowing direct access to the tubal lumen and ovarian surface.

For the configuration in which the reproductive tract was kept intact, a porcine insemination catheter was first guided through the opening of the uterus into the opening of the Fallopian tubes to then feed the endoscope prototype through it to start imaging in the proximal portion of the tubes. Because the Fallopian tubes collapse naturally once removed from the body, a pressurized air source was used to inflate the tubes before imaging.

In the isolated structures configuration, a 3D-printed support piece was placed at the proximal end of the tube, providing both an air inlet for insufflation and a channel through which the endoscope was inserted into the lumen. The endoscope entered from the proximal (uterine) side, advancing toward the fimbria and ovary. The ovary at the distal end acted as a natural seal that prevented air from escaping and maintained sufficient distension throughout the procedure. The tube was positioned horizontally and stabilized by pinning the mesosalpinx to a supporting base.

#### Signal-to-Noise Radio (*SNR*) Acquisition

To determine how image quality varies with the illumination intensity across a range of clinically relevant operating conditions, the *SNR* was characterized as a function of the µLED drive current using one of the ex vivo porcine Fallopian tube samples. The prototype was positioned inside the lumen, and the drive current was swept from 1 to 20 mA, with the exposure time adjusted at each step to maintain image usability while keeping gain fixed at 1 and offset at 0 to eliminate automatic gain artifacts. At each current level, nine frames were acquired and averaged pixel-by-pixel to reduce shot and readout noise. A region of interest (ROI) was manually selected within a homogeneous area of mucosal tissue, avoiding specular reflections and tissue boundaries, and the *SNR* was computed as:

(6) $$SNR=20\log_{10}\left(\frac{\mu }{\sigma }\right)$$

where *µ* is the mean, and *σ* is the standard deviation of the pixel intensity values within the selected ROI.

## 3. Results and Discussion

### 3.1. Illumination System

#### 3.1.1. SPD, CCT and CRI

**Figure 13:**
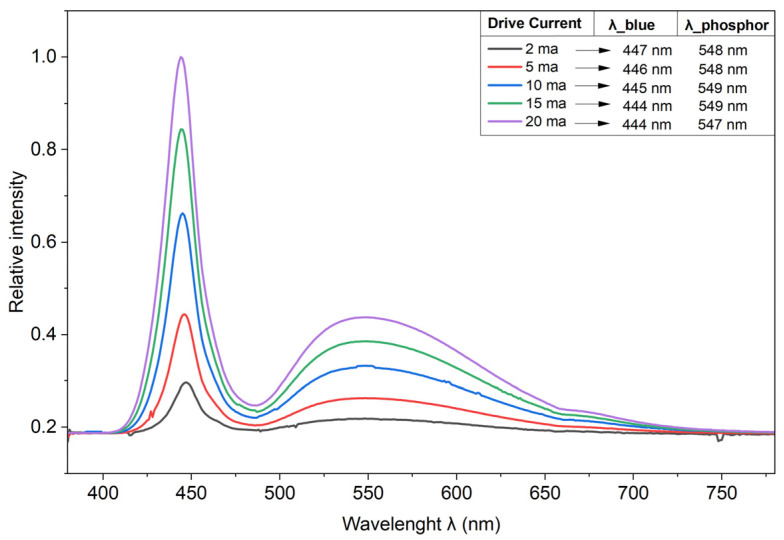
Normalized SPD of the µLED at five drive currents.

The µLED SPD is shown in Figure 13 for the five drive currents tested. The spectrum exhibits the typical shape of a phosphor-converted white LED, with a narrow blue peak around 445 nm followed by a broad emission band centered around 550 nm. This result is consistent with the relative intensity versus wavelength curve provided in the manufacturer’s datasheet. The blue peak position remained essentially unchanged across the explored current range, with no measurable shift, indicating that the junction temperature was stable enough to prevent the wavelength drifting toward longer wavelengths, as typically observed in InGaN emitters under thermal loading.

**Figure 14:**
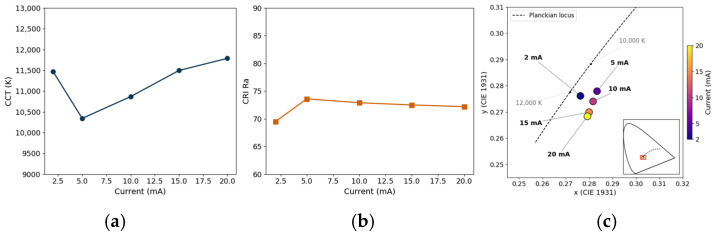
µLED chromatic characterization: (**a**) CCT; (**b**) CRI Ra; (**c**) CIE 1931 chromaticity.

The chromatic properties derived from the measured spectra are summarized in Figure 14. Across the explored operating range, the µLED exhibits a consistently high CCT, varying from 10,343 K at 5 mA to 11,788 K at 20 mA (Figure 14a). At the same time, the CRI remains between 69.5 and 73.6 (Figure 14b). In the CIE 1931 chromaticity diagram (Figure 14c), all measured points lie below the Planckian locus in the cool-blue region, with only small shifts in chromaticity coordinates, from (0.276, 0.276) at 2 mA to (0.279, 0.268) at 20 mA.

The proposed endoscope is intended primarily for navigation and visualization inside narrow anatomical cavities such as the Fallopian tube, where diagnostic information relies more on lumen morphology and vascular visibility than on precise color discrimination. The measured CCT differs from the values typically targeted in surgical illumination systems, which generally favor CCTs between 3000 K and 6700 K to ensure a natural color appearance. However, these requirements are defined by IEC 60601-2-41, which explicitly excludes endoscopes and their light sources from its scope [36,37]. In endoscopy, higher CCTs are in fact often preferred, since a cooler spectrum tends to provide sharper images of the inspected cavity [36]. The strong blue component of the µLED further suggests its potential for this application, since it falls near the hemoglobin absorption band exploited in narrow-band imaging to enhance the visibility of superficial mucosal vasculature [38].

#### 3.1.2. LIV

**Figure 15:**
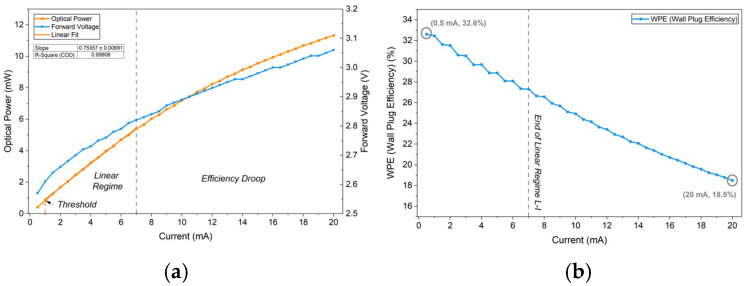
Electrical and optical characterization of the µLED: (**a**) light-current-voltage (LIV) curves; (**b**) wall-plug efficiency (WPE) as a function of drive current.

The LIV curve of the µLED, shown in Figure 15a, shows that the optical output power increases monotonically with the drive current. Between 1.0 and 7.0 mA, the L-I response can be described by a linear fit with slope efficiency of 0.7536 ± 0.0069 mW/mA ($R^{2}>0.999$), above which the rate of increase begins to slow, consistent with the onset of efficiency droop in nitride-based LEDs, and the WPE falls from a peak of 32.6% at 0.5 mA to 18.5% at 20.0 mA, as shown in Figure 15b.

#### 3.1.3. Irradiance

**Figure 16:**
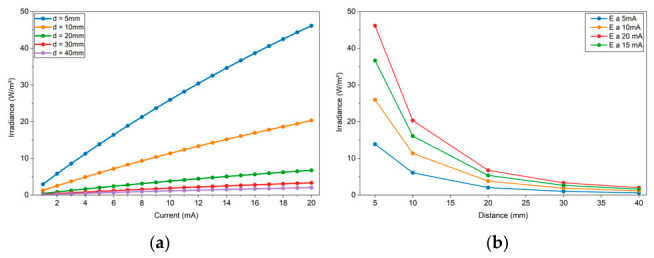
Measured irradiance at the distal tip as a function of (**a**) drive current and (**b**) working distance.

The irradiance shown in Figure 16 increases proportionally with drive current at all working distances, ranging from 2.95 to 46.16 $W/m^{2}$ at 5.0 mm and from 0.13 to 2.01 $W/m^{2}$ at 40.0 mm, confirming that the illumination level can be adjusted predictably by controlling the drive current and working distance. As expected, the irradiance decreased with working distance, falling by approximately 85% between 5.0 and 40.0 mm across the full current range, though the narrow dimensions of the target cavities naturally constrain the working distance to the shorter end of this range, where illumination is most effective.

**Figure 17:**
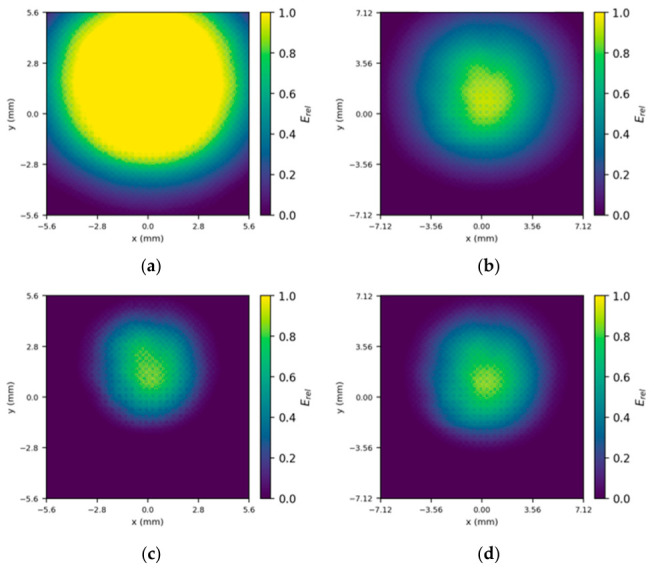
Two-dimensional relative irradiance maps of the µLED illumination acquired using a Spectralon diffuse reflectance standard, at distances of (**a**) 4 mm and (**b**) 6 mm with fixed gain, and (**c**) 4 mm and (**d**) 6 mm with automatic gain.

Figure 17 presents the two-dimensional irradiance maps obtained from the Spectralon imaging at working distances of 4 mm and 6 mm. Under fixed-gain conditions, the CV was 0.40 at 4 mm and 0.60 at 6 mm. In both cases, the irradiance distribution exhibits a characteristic intensity gradient along the sensor-µLED axis, with the highest irradiance observed closest to the µLED and a gradual decrease towards the opposite edge of the FOV. The lower CV at 4 mm reflects the shorter working distance, at which the wide emission angle of the µLED (120°) produces a better spatial overlap between the illumination cone and the sensor FOV. As the working distance increases to 6 mm, the illumination footprint expands, and the angular emission profile of the µLED becomes more pronounced, leading to greater peripheral irradiance attenuation and, consequently, increased spatial non-uniformity.

Under automatic gain control, the measured CV was 0.56 at both 4 mm and 6 mm. The reduced distance dependence is attributable to the sensor’s brightness compensation, which partially compensates for peripheral irradiance attenuation and produces a more homogeneous recorded intensity distribution. These values confirm that the fixed-gain condition provides a more representative characterization of the intrinsic illumination distribution, while the automatic gain mode masks part of the spatial non-uniformity through its adaptive response.

### 3.2. Imaging System

#### 3.2.1. FOV

Endoscopes typically require a wide field of view to compensate for the limited range of movement inside anatomical cavities, allowing the user to capture as much information as possible in a single image without repositioning the device. The system achieved a mean diagonal FOV of 115.0° ± 2.4°, with mean horizontal and vertical components of 93.9° ± 4.5° and 97.8° ± 1.0°, respectively. These values are consistent with the wide-angle imaging typically targeted in endoscopic systems and comparable with the ~45° reported by Cordova et al. [35] for a Fallopian tube endoscope with a different optical architecture. Additionally, they are consistent with the 120° nominal emission angle of the integrated µLED, confirming that the illumination and imaging systems are well matched in angular coverage.

Alongside the FOV, the level of detail the system can capture is equally important, as clinically relevant features such as mucosal texture and superficial vasculature require sufficient spatial resolution to be distinguished.

#### 3.2.2. MTF

**Figure 18:**
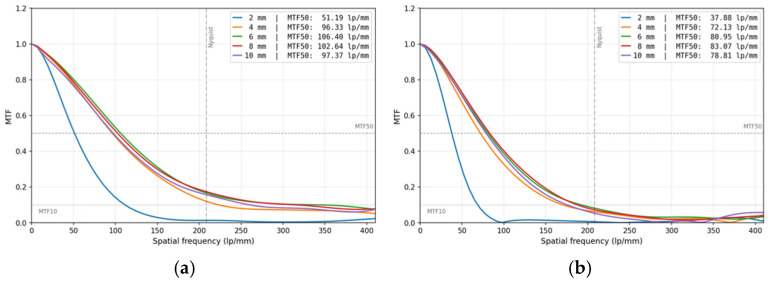
MTF curves as a function of spatial frequency for working distances ranging from 2 to 10 mm: (**a**) automatic gain; (**b**) fixed gain (Gain = 2, Offset = 2, Exposure = 160 µs).

The MTF curves obtained across working distances from 2 to 10 mm are presented in Figure 18.

The analysis of the slanted-edge MTF curves under fixed gain (Gain = 2, Offset = 2, Exposure = 160 µs) yielded a peak MTF50 of 83.07 lp/mm (0.1994 cy/px) at 8 mm working distance. Across the 4–10 mm range, the MTF50 remained above 72 lp/mm, indicating consistent resolving capability throughout the expected operating distances during Fallopian tube navigation. At 2 mm working distance, the MTF50 dropped to 37.88 lp/mm (0.0909 cy/px), consistent with the onset of defocus below the 4 mm minimum of the designed depth of field reported in the NanEyeM datasheet. Under automatic gain, the peak MTF50 reached 106.40 lp/mm (0.2553 cy/px) at 6 mm working distance, and remained above 96 lp/mm between 4 and 10 mm. For both gain conditions, the MTF10 approached the Nyquist limit of the sensor (208.33 lp/mm, 0.5 cy/px) across the 4–10 mm range.

The MTF50 values obtained under automatic gain were consistently higher than those measured under fixed gain across all working distances. Wang et al. [27] demonstrated that automatic gain control can compromise the linearity of pixel values and introduce artefacts that alter the measured MTF, with the magnitude of the effect depending on the specific system and acquisition conditions, which may partly explain the systematic difference observed between the two gain conditions.

The peak MTF50 of 0.2553 cy/px under automatic gain at 6 mm working distance is comparable in order of magnitude to the 0.17 cy/px reported by Slomka et al. [39] for a miniaturized endoscope based on a 320 × 320-pixel CMOS sensor with 2.4 µm pixel pitch at 10 mm working distance, with the remaining difference attributable to the distinct lens configurations and working distances at which peak performance was observed in each system.

### 3.3. Mechanical Performance and Safety

#### 3.3.1. Thermal Assessment

**Figure 19:**
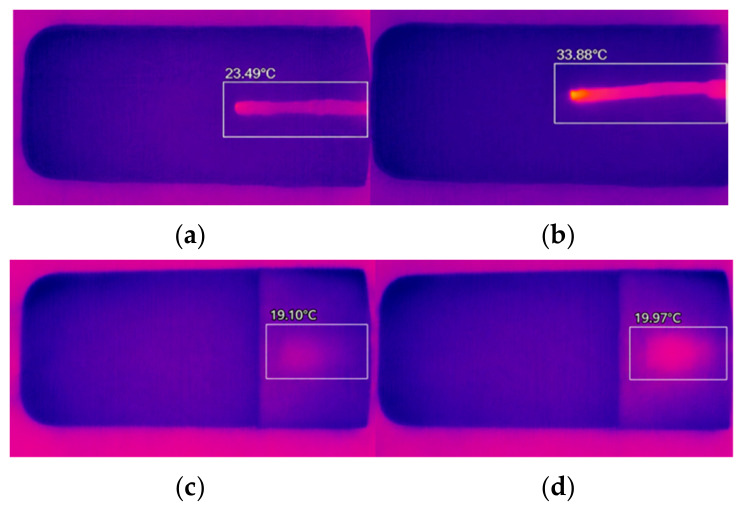
Infrared images captured during the thermal characterization of the tissue-mimicking phantom. (**a**,**b**) correspond to the configuration with the prototype placed on the surface of the phantom at µLED drive currents of 5.0 mA and 20.0 mA, respectively, and (**c**,**d**) correspond to the configuration with the prototype inserted into the phantom at µLED drive currents of 5.0 mA and 20.0 mA, respectively.

For the surface-contact configuration, the prototype reached steady-state temperatures of 23.49 °C and 33.88 °C for µLED currents of 5.0 mA and 20.0 mA, respectively. In the inserted configuration, lower temperatures were observed, with values of 19.10 °C at 5.0 mA and 19.97 °C at 20.0 mA. The infrared thermal images captured during the measurements are presented in Figure 19.

The reduced temperatures observed in the inserted configuration are likely attributed to the larger contact area between the prototype and the agar phantom, promoting more efficient heat dissipation through the surrounding material. In contrast, the surface configuration has less tip contact with the phantom, resulting in less thermal dissipation through the agar, and thus increased localized temperature accumulation around the prototype tip.

Overall, the results show that the proposed illumination system operates within temperature ranges suitable for short-term contact with tissue environments, especially at the typical drive current of 5.0 mA. Despite increased heating at the maximum tested current of 20.0 mA, the measured temperatures remained below those commonly associated with immediate thermal damage in biological tissue during brief exposure periods. This result is in accordance with the International Electrotechnical Commission IEC 60601-1 standard, which dictates a safety limit of 43 °C [40]. These findings indicate that the reduced dimensions of the chosen µLED, combined with its low-power operation and chosen encapsulation approaches, help to limit thermal accumulation at the distal end of the prototype, thereby supporting its use in miniature endoscopic systems designed for confined anatomical environments.

#### 3.3.2. Mechanical and Steering Performance

**Figure 20:**
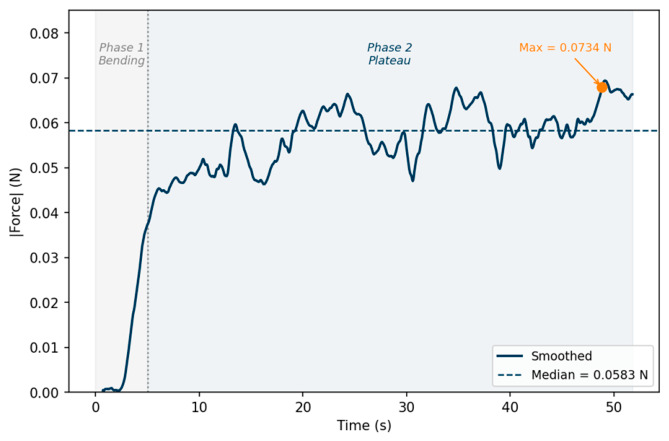
Tip force of the prototype as a function of time.

The tip force of the prototype is presented in Figure 20 as a function of time.

The force profile obtained during distal tip actuation exhibits two distinct phases: an initial bending phase associated with the progressive tensioning of the pull wire, followed by a plateau region corresponding to the maximum attainable force after complete tip deflection. The plateau was reached after approximately 5 s of pulling, with a maximum measured contact force of 0.0734 N. Based on visual analysis of the contact geometry during testing, the applied force was estimated to be distributed over approximately one-third of the distal tip cross-sectional area, corresponding to 0.8482 mm^2^. From this contact area, a maximum local pressure of approximately 86.6 kPa (12.55 psi) was calculated.

The obtained pressure values remain below the safety thresholds reported in the literature for Fallopian tube manipulation, namely 30 psi for the proximal and middle regions and 15 psi for the distal region [41]. These results indicate that the steering mechanism generates adequate actuation force for directional navigation while maintaining pressure levels safe for delicate tubular tissues, such as the Fallopian tubes.

**Figure 21:**
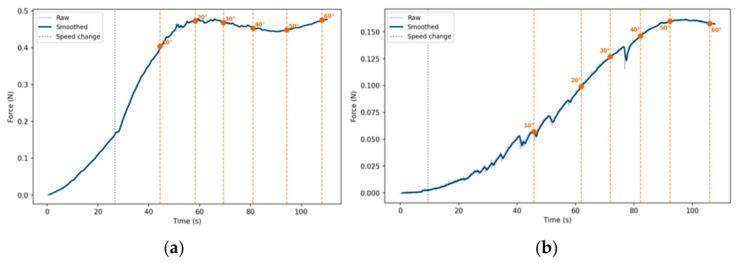
Measured traction force for (**a**) fully assembled prototype, (**b**) prototype with only the steering pull-wires assembled.

For the traction force measurements, they revealed that the integrated electrical interconnections have a major influence on the mechanical behavior of the prototype. In the fully assembled configuration, as observed in Figure 21a, which included the CMOS camera wiring and µLED electrical wiring, the traction forces rose to approximately 0.48 N at a 20° deflection angle. Beyond this deflection, the force plateaued despite further angular displacement, likely due to the steering wire cutting through the flexible encapsulation material during testing, thereby reducing mechanical resistance. Therefore, only data up to 20° was used for comparative analysis between different configurations.

Post-test inspection revealed that the steering wire had partially cut through the flexible encapsulation, due to cumulative mechanical wear from previous assembly and characterization procedures, combined with the overload conditions imposed during this destructive test. This failure occurred beyond the intended operating range of the prototype and was therefore considered representative of its mechanical limit rather than its expected performance under normal operating conditions.

The prototype with only the steering wire (as observed in Figure 21b), however, required about 0.11 N to achieve the same 20° deflection. This significant reduction in the required traction force highlights that the additional wiring of the camera and the µLED affects the overall stiffness of the distal structure and thus increases its resistance to bending. These findings highlight the mechanical effect of electrical integration in ultra-miniaturized endoscopic systems and emphasize the importance of optimizing wire routing and encapsulation geometry to maintain steering performance while ensuring device functionality.

**Figure 22:**
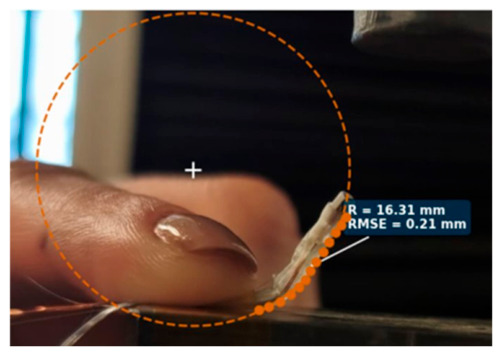
Image of the prototype’s bending radius at full deflection.

The curvature analysis at maximum actuation, as depicted in Figure 22, resulted in a bending radius of 16.31 mm, with a root-mean-square fitting error of 0.21 mm, indicating good agreement between the experimental curvature profile and the fitted circular model. In addition, the prototype could achieve bidirectional steering angles of about 60°. These findings are consistent with the dimensional and navigational requirements reported for Fallopian tube exploration by Cordova et al. [35], who state that bending radii of less than 30 mm are desirable for following the anatomical curvature of the tube, while steering capabilities of 60° are advantageous for visualizing the fimbria and distal tubal regions. Single-axis deflection combined with axial shaft rotation has been demonstrated as a sufficient navigation strategy for this anatomy, having been consistently adopted across successive generations of steerable falloposcopes [35,42,43].

The performance of the present prototype can be compared with representative miniaturized endoscopic systems reported in the literature, as summarized in Table 2 [35,39,44,45,46].

### 3.4. Ex Vivo Validation

The prototype was validated using ex vivo porcine reproductive tissue under the two configurations described in Section 2.8. All images presented were extracted from real-time video streams acquired at a frame rate of approximately 20 Hz during the procedure.

**Figure 23:**
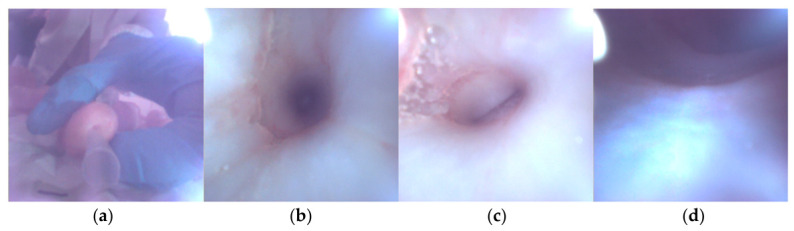
Representative frames from the first ex vivo test with automatic gain, offset and exposure time, and frame rate of 20 Hz: (**a**) prototype tip outside the reproductive tract; (**b**) uterine tissue surrounding the tubal opening; (**c**) transition toward the Fallopian tube lumen; (**d**) inside the Fallopian tube.

In the first test, images were acquired with the camera’s automatic gain control active. Figure 23 presents four representative frames: one showing the prototype tip approaching the reproductive tract, and three acquired progressively inside the system, including the transition toward the Fallopian tube lumen, where air bubbles are visible, and a final frame already inside the tube where some mucosal folds can be detected. Tip deflection was successfully achieved during navigation, confirming the operation of the steering mechanism within the lumen. Some localized overexposure was observed in parts of the images, which is expected under automatic gain control in close-range intraluminal conditions.

**Figure 24:**
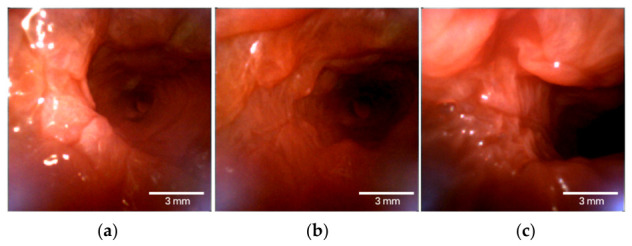
Representative frames from the second ex vivo test with fixed gain: (**a**) isthmus; (**b**) ampulla; (**c**) infundibulum. These images were acquired with an exposure time of 120 ms, frame rate of 20 Hz and analog gain of 1 dB. The scale bar on each figure was estimated from the average diameter of the porcine Fallopian tube and does not represent a direct spatial calibration.

In the second test, automatic gain was disabled, and a fixed gain value was applied instead. Images were obtained in three main regions of the Fallopian tube: isthmus, ampulla, and infundibulum, as shown in Figure 24. Compared to the first test, color rendition improved noticeably, with the pinkish tone of the mucosal tissue more faithfully represented. In the isthmus, superficial vascular structures are distinguishable as linear patterns of slightly darker tone against the surrounding tissue. In the ampulla, longitudinal mucosal folds and polypoid-like projections are visible, consistent with the plicae characteristic of this region. In the infundibulum, the luminal opening is surrounded by mucosal tissue with a more irregular surface, reflecting the transition toward the fimbria region.

In both tests, and in all of the four independent porcine Fallopian tube samples that were used, the prototype was successfully advanced through the tubal lumen up until its maximum insertable length of 40 cm, with visualization of the proximal, medial and distal segments, as seen in Figure 23 (isthmus, ampulla and infundibulum). Mucosal folds, granular tissue texture and regional anatomical differences were consistently identified in all samples, whereas superficial vascular structures were only occasionally observed, likely due to the use of previously frozen and thawed tissue. No macroscopic tissue damage was detected following the imaging procedures. The color shift observed in the first test was largely resolved by switching to a fixed gain, confirming that image quality in this system is sensitive to acquisition parameter configuration. Some bright reflections remain visible at the image periphery in the second test, likely due to a slight displacement of the camera relative to the illumination module.

Image quality improved with increasing current up to 5 mA, where the *SNR* reached a maximum of 34 dB. The *SNR* remained stable in the 5–8 mA range and then gradually decreased at higher currents, as exposure times were reduced to compensate for increased irradiance and to prevent sensor saturation. These results suggest 5 mA with an exposure of 120 µs as the combination that best balances illumination intensity and noise performance in ex vivo tissue.

## 4. Conclusions

This work demonstrated the feasibility of a 1.8 mm chip-on-tip endoscopic prototype that integrates a CMOS image sensor and a single µLED to visualize narrow anatomical cavities. The optical system delivered a diagonal field of view of 115.0° ± 2.4° and a peak spatial resolution of 83 lp/mm, enabling the discrimination of mucosal features relevant to Fallopian tube exploration. The illumination system achieved a minimal CCT of 10,343 K and a maximal CRI of 73.6, and the distal tip temperature remained below 43 °C across all tested operating conditions.

The ex vivo validation using porcine reproductive tract tissue confirmed that the system acquires images with the morphological detail required to distinguish the main anatomical regions. For a first proof-of-concept prototype, these results support the feasibility of sub-2 mm chip-on-tip endoscopy for this application. Image acquisition parameters still require optimization to ensure consistent image quality across varying intraluminal conditions, and fabrication and assembly procedures can still be improved to better support interactive development.

The transition from proof-of-concept to a clinically viable device will additionally require formal biocompatibility evaluation of all patient-contacting materials in accordance with the ISO 10,993 standard, electrical safety characterization including leakage current assessment, and validation of material compatibility with the pre-use terminal sterilization process adopted during manufacturing.

Although developed with gynecological applications in mind, the same platform could be adapted to other anatomical environments where access is constrained by narrow luminal dimensions, such as the biliary ducts, urinary system, or neurosurgical pathways.

## Figures and Tables

**Table 1:** Duration needed for fabrication and assembly of the different prototype components.

	Printing Duration	Washing and Curing Duration	Assembly Duration
Imaging and illumination module	3 h 2 min	10 min	30 min
Steering module	20 min	10 min	2 min
Flexible tube	55 min	10 min	-----
Final prototype	-----	-----	30 min

**Table 2:** Comparative performance characteristics of representative miniaturized endoscopic systems in relation to the present prototype.

System	OD (mm)	Imaging	FOV	Resolution	Illumination	Steering	Validation	Application
This Work	1.9	CMOS (320 × 320 px)	~115.0	83 lp/mm @WD of 8 mm	µLED on-tip	Single-axis, 60°	Ex vivo. porcine	Fallopian tube
Cordova et al. [35]	0.79	Fiber bundle	~45°	88 µm @WD of 5 mm	External LED + fiber	Single-axis, Unidirectional	Ex vivo, porcine	Fallopian tube
Buenconsejo et al. [44]	0.9	Single double-clad fiber	helical scan, up to 5.65 × 140 mm^2^	25 µm	External RGB laser + fiber	n.r	Ex vivo, tongue and finger	Small- diameter luminal organs
Slomka et al. [39]	2.7	CMOS (320 × 320 px)	~60°	71 lp/mm @WD of 10 mm (MTF-derived)	External LEDs + fiber	n.r	Ex vivo, murine GI	Gastrointestinal tract
Boese et al. [45]	5.6	HD CMOS	n.r	n.r	LED on-tip	Swiveling tip, 100°	Bench	Upper airways
Cheng et al. [46]	8.5	CMOS (1000 × 1000 px)	~131°	~13 lp/mm @WD of 10 mm	LED on-tip	n.r	Bench	spine

## Data Availability

Data is contained within the article.

## Abbreviations

The following abbreviations are used in this manuscript:

ADC	Analog-to-digital converter
CCD	Charge-coupled device
CCT	Correlated color temperature
CMOS	Complementary metal-oxide semiconductor
CRI	Color Rendering Index
CRI Ra	General Color Rendering Index
ESF	Edge spread function
FOV	Field of view
IPA	Isopropyl alcohol
LIV	Light-current-voltage
LSF	Line spread function
LVDS	Low-voltage differential signaling
µLED	Micro light-emitting diode
MTF	Modulation transfer function
SNR	Signal-to-noise radio
SPD	Spectral power distribution
UV	Ultraviolet
WPE	Wall-plug efficiency
